# An exploration of the differences in hip strength, gluteus medius activity, and trunk, pelvis, and lower-limb biomechanics during different functional tasks

**DOI:** 10.1080/23335432.2020.1728381

**Published:** 2020-02-17

**Authors:** Komsak Sinsurin, Raul Valldecabres, Jim Richards

**Affiliations:** a Biomechanics and Sports Research Unit, Faculty of Physical Therapy, Mahidol University, Nakhon Pathom, Thailand; b PhD School of Catholic University of Valencia, Spain; c Allied Health Research Unit, University of Central Lancashire, Preston, UK

**Keywords:** Pelvic drop, lateral trunk bending, step down, joint moment, muscle activity, walking

## Abstract

The purpose of the study was to explore differences in the coronal biomechanics of the trunk, pelvis, hip, and knee joints, and gluteus medius muscle activity (GMed) during walking and step down from two riser heights. Joint kinematics and kinetics from 20 healthy participants were recorded using a 10-camera Qualisys system and force plates, and GMed EMG was recorded using a Delsys Trigno system. Hip abductor strength was measured using a hand-held dynamometer. Pelvic obliquity and lateral trunk bending excursions were significantly higher in walking than in step-down tasks. Significantly greater knee adduction moments were seen during both step-down tasks compared to level walking with significantly greater GMed activity. However, a significant interaction between side and task was seen for hip adduction moment, with step-down tasks showing lower hip moments than during walking, with greater peak hip moments being more apparent in the dominant limb. This suggests the GMed has a greater stabilizing role during the step-down tasks, although walking required a greater mechanical demand. Health professionals should expect to find less excursion of lateral trunk bending in step-down tasks compared to level walking and consider that GMed has different roles in these two tasks.

## Introduction

Walking and step tasks are commonly used in activities of daily living and during rehabilitative programs. Biomechanical data from healthy individuals is crucial as this provides baseline information in order to determine clinically important differences between healthy subjects and people who may be at risk of injury. Coronal plane movements of the trunk, pelvis, and lower-limb joints have been identified as key factors which contribute to the development of knee problems, such as patellofemoral pain (PFP) (Powers 2010), non-contact ACL injuries (Hewett et al. 2009), and knee osteoarthritis (OA) (Chang et al. 2005). Asymmetries and greater movements are often noted and associated with clinical impairment, such as lateral trunk bending, pelvic drop, and knee valgus, and have been identified as a risk factor of injury development (Zhang et al. 2008). Powers (2003) identified that compensation of ipsilateral trunk bending in PFP contributed to an increase in knee abduction moment and greater stress on the patellar femoral joint. Such trunk compensations have also been identified in people with knee OA (Tanaka et al. 2008), with greater knee adduction moments being linked to greater loading on the medial compartment of the knee.

The hip abductor muscles have been identified as the most important muscles to control and stabilize the pelvis during locomotion (Widler et al. 2009). The Gluteus Medius (GMed) muscle plays a key role in controlling the coronal pelvic motion (Flack et al. 2014) and any functional impairment of this muscle can lead to excessive lateral trunk bending, which can be observed in people with a variety of knee problems including knee OA (Chang et al. 2005) and PFP (Dierks et al. 2008). Chang et al. (2005) proposed that coronal plane movement impairment in people with GMed weakness includes ipsilateral trunk bending and contralateral pelvic drop during stance, which is also a mechanism that increases the loading on the medial compartment of the knee. Therefore, this could be a major contributing factor in the development of genu varum. However, the role of the GMed muscle function during different movement tasks has not been fully explored. The coronal plane biomechanics of the trunk, pelvis, hip, and knee joints, and GMed muscle function data would be useful to enable a better understanding of movement control and for consideration in clinical practice during functional tasks or activities of daily living.

Functional tasks are frequently used in the assessment of knee problems in clinical practice to determine if abnormal movements exist, these include; level walking, step up, step down, and stair climbing. However, little or no data exists quantifying hip abductor strength, GMed activity, and the coronal plane biomechanics of the trunk, pelvis, hip, and knee during these tasks. To have baseline data of the coronal plane movements and hip abductor strength in healthy adults,  this study explored differences exist in the clinical assessment of hip strength, GMed muscle activity, and coronal plane biomechanics of the trunk, pelvis, hip, and knee and whether differences exist between three clinical assessment tasks; walking, a 20 cm step down and 30 cm step down. Our hypotheses were that significantly greater movements in biomechanical parameters would be seen during step down compared to walking, and the greater the step height the greater the demand. Any side-to-side differences in the clinical hip strength assessment would be apparent in the biomechanical data and may indicate different strategies between the different tasks. These findings may increase our understanding of the interaction between trunk and pelvic control and hip and knee joint biomechanics during walking and step-down tests. This could help clinical professionals by providing reference values on the typical movement strategies and GMed activity and their association with hip strength, which would be useful when considering assessment during rehabilitation programs.

## Methods

### Participants

Twenty healthy participants were included in the study. Participants were excluded if they had any current musculoskeletal injuries or disorders, history of surgery or traumatic injury to the lower extremities, current or a history of medical conditions that affect movement. All participants signed a consent form before testing, and the study was approved by the University of Central Lancashire Ethics committee.

### Procedures

Electromyography data was recorded using surface EMG sensors attached over both GMed muscles using Trigno EMG sensors sampling at 2000 Hz (Delsys Inc, USA) and force data was collected using 4 AMTI BP400600 force plates at 1000 Hz (AMTI, USA), which were synchronized with a 10-camera Oqus 7 motion capture system sampling at 200 Hz (Qualisys, Gothenburg, Sweden). Twenty-two reflective markers were attached over the bony prominences of both sides including; acromion process, anterior superior iliac spine (ASIS), posterior superior iliac spine (PSIS), lateral and medial femoral epicondyle, lateral and medial malleolus, distal head of the first metatarsal, distal head of the fifth metatarsal, proximal head of the fifth metatarsal, and heel. In addition, five clusters of 4 markers were placed on the spinous process of 10th thoracic spine, lateral thigh, and lateral shank. Before EMG sensor placement, alcohol wipes were used to clean and reduce skin resistance over the target area. The European Recommendations for Surface Electromyography (SENIAM) were used to determine the location of the EMG sensors.

#### Walking and step-down tests

Participants were asked to stand in the middle of the capture space and a static anatomical trial was recorded. Participants were then asked to perform walking and step tasks, respectively. Four step-down tasks were performed; non-dominant limb (NDL) and dominant limb (DL) from 20 to 30 cm step heights in a randomized order. The lower-limb dominance was determined as the preferred leg to kick a ball for maximal distance (Distefano et al. 2009).

All participants were asked to walk at their usual speed, for the step-down tasks, participants were asked to stand on two heights of wooden step and step down bringing both feet together on the bottom step with comfortable speed. Five successful trials from each side and each step height were recorded.

#### Clinical hip abductor strength and maximum voluntary isometric contraction (MVIC) test

**Figure 1.**
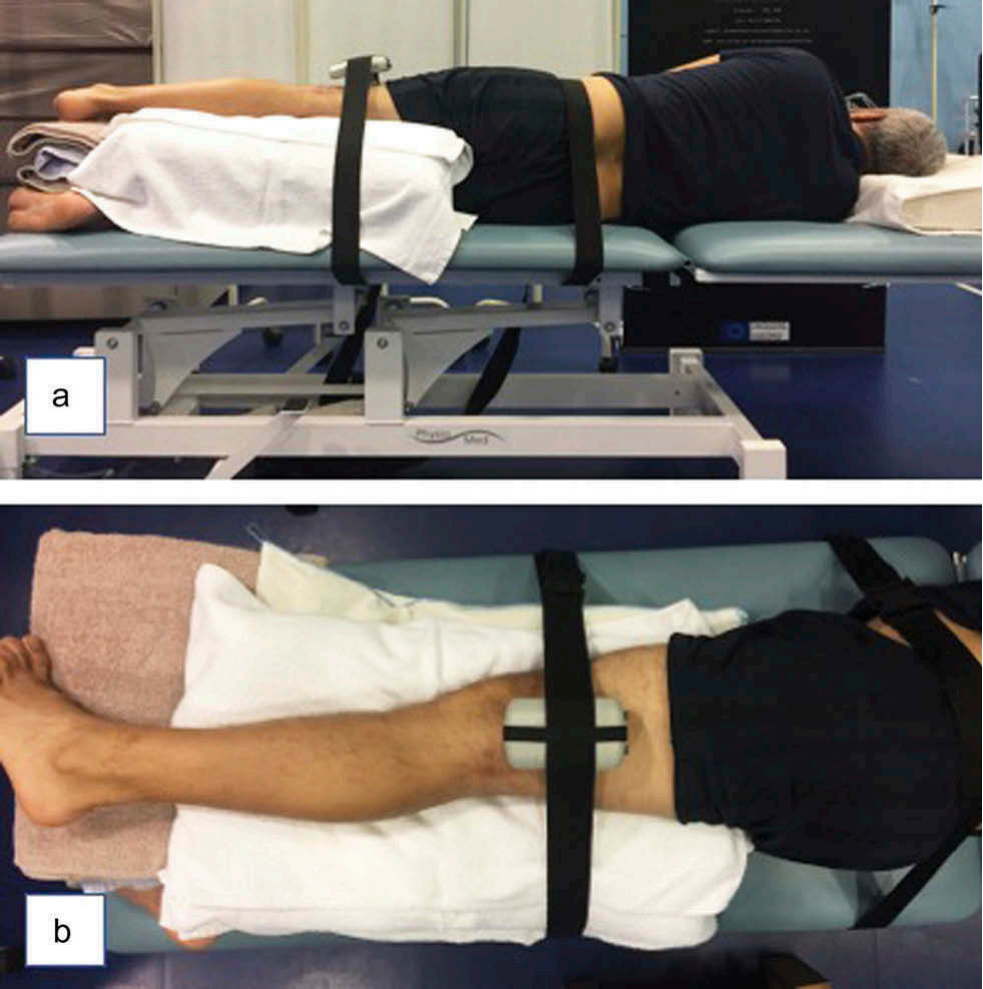
Hip abductor strength test. (a) posterior view, (b) superior view. The participant was in a side-lying position with the tested leg uppermost in the neutral position. The dynamometer mounted against a stabilization strap was held perpendicular to the leg

After the walking and step-down tasks, the participants’ hip abductor strength was tested using a commonly used clinical method (Kim et al. 2018). The participant was positioned in a neutral anatomical side-lying position with the tested leg being above the contralateral limb (Figure 1). The participants were then asked to abduct their leg with maximum force for 3 s sideways, i.e. towards the ceiling, pushing against a hand-held dynamometer (Model 01165, Lafayette Instrument Company, USA). A hand-held dynamometer is an inexpensive piece of equipment, which can provide good reliability, and has previously been shown to have a moderate to high correlation with isometric dynamometers (Arnold et al. 2010). To ensure the abductor muscle strength was tested and the lower limb did not rotate externally, the participants were instructed to ensure their toes were pointed horizontally during the contraction. The force exerted in this position against the dynamometer was recorded and the moment arm of the adduction/abduction axis of the hip joint was measured using a tape measure from the level of the dynamometer to the greater trochanter. These measurements were used to calculate the maximum hip abductor strength (Nm) during an MVIC test as the product of the force (N) and moment arm (m). EMG data was also recorded during the strength test which was then used to normalize all EMG signals to the maximum voluntary contraction. Three repetitions of the strength test were measured for each side and the order of NDL and DL strength testing was randomized, with at least 30 s rest between trials.

#### Data analysis

Marker coordinates, ground reaction force, and EMG data were exported from Qualisys Track Manager to Visual 3D software. A three-dimensional model was constructed in Visual 3D (C-Motion, Rockville, MD, USA). Marker coordinates and force data were filtered using a second-order zero-lag Butterworth digital filter with cut-off frequencies of 6 Hz and 40 Hz, respectively. The static anatomical trial was captured in static standing and the data was used to define and set up the link model of bony segments including the pelvis, thigh, lower leg, and foot segments. This was then applied to the motion trials.

The segment coordinate system of the pelvis was defined by the ASIS and PSIS markers, from which the hip joint center was calculated automatically (Bell et al. 1990) when applied V3D_Composite pelvic segment. The thigh segment coordinate system was defined by the hip joint center and the medial and lateral femoral epicondyles, the shank coordinate system was defined by the medial and lateral femoral epicondyles and medial and lateral malleolus, and the foot coordinate system was defined by the mid-point between the medial and lateral malleolus and the mid-point between the distal head of the first metatarsal and distal head of the fifth metatarsal.

For the walking trials, the stance phase was defined by heel strike to heel strike, and for the step-down trials the stance phase was defined between the toe-off of the contralateral foot to the initial contact on the lower step. The average values from the successful trials for each limb and each step height were reported and analyzed. Contralateral pelvic drop angle, pelvic obliquity excursion angle, lateral trunk bending excursion angle, and peak hip and knee adduction moments (Nm/kg) were reported and compared between each limb and task.

EMG data from each muscle was collected with a bandpass frequency of 20 Hz to 450 Hz and then full-wave rectified. In addition, a 500 ms window EMG of MVIC test was selected for normalization purposes and the average EMG during stance phase was reported as a percentage of MVIC. EMG data from one participant was incomplete. Therefore, the EMG findings were reported from 19 participants.

Statistical analysis was performed using SPSS version 17. All kinetic and kinematic data were examined for normality and found suitable for parametric testing. Statistical significance was set at an alpha level of p ≤ 0.05. Paired T-tests were used for statistical comparison of hip abductor strength between limbs and two factor (2 × 3) repeated measure ANOVA tests were conducted to explore the effect of limbs and tasks and the interactions between these factors. Where significant main effects were seen, post hoc pairwise comparisons were performed. Average GMed EMG data was found to be non-normally distributed; therefore, a Friedman test was used to analyze the effect of task and individual comparisons were performed using Wilcoxon Signed Ranks tests.

## Results

**Figure 2.**
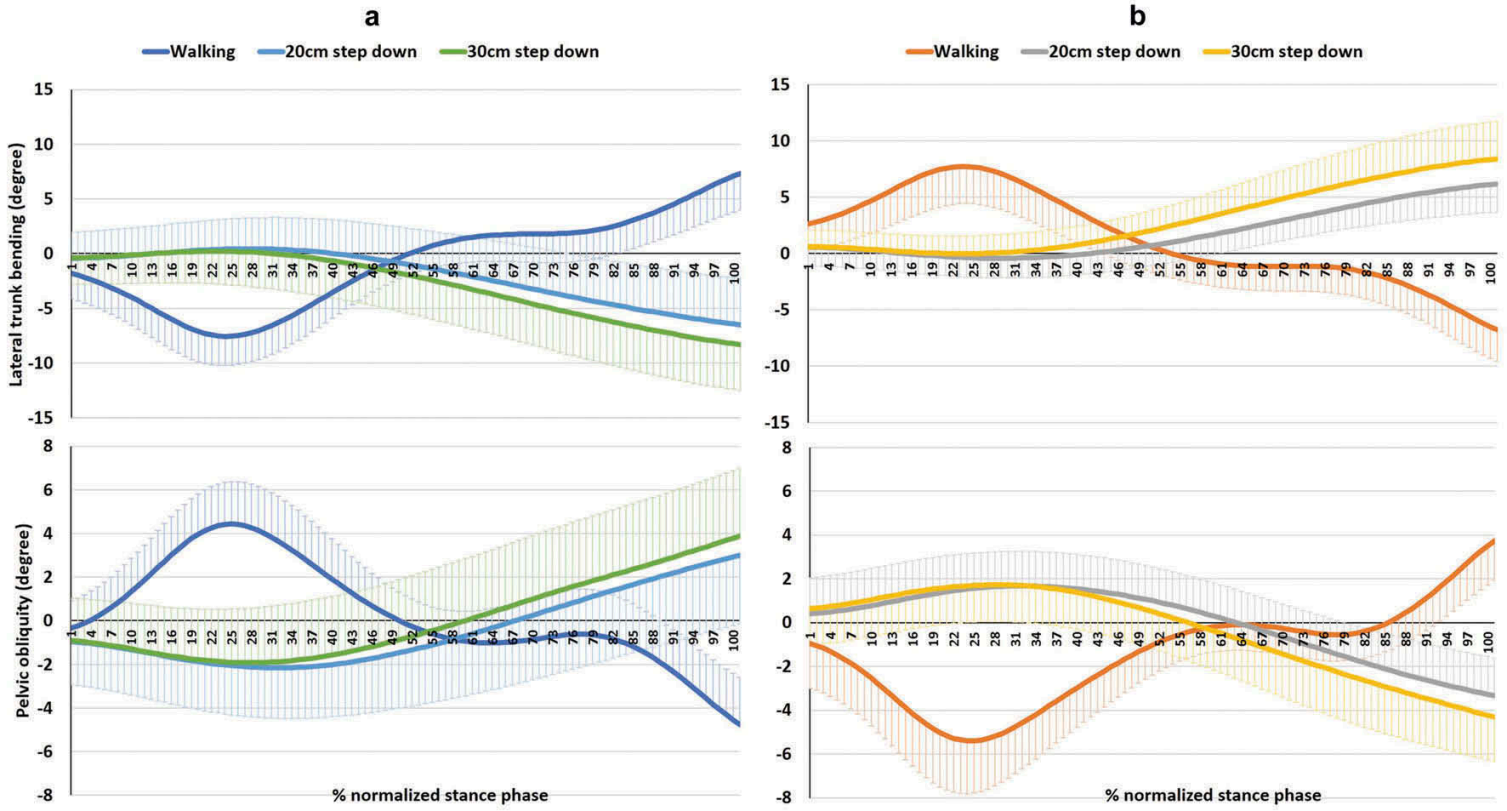
Pelvic obliquity and lateral trunk bending during the stance of both limbs walking and step-down tasks. (a) and (b) columns are for the NDLs and DLs, respectively. In Y-axis, positive and negative represent the direction to right and to left, respectively

**Figure 3.**
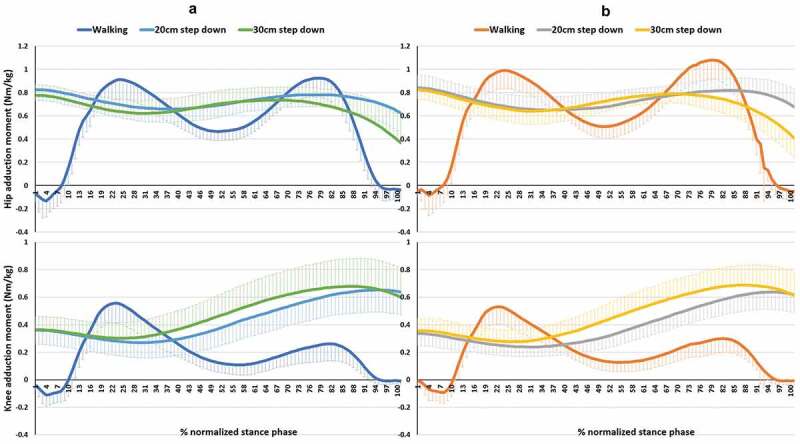
Hip and knee adduction moment of both sides during walking and step-down tasks. (a) column is for the NLs. (b) column is for DLs. In Y-axis, positive and negative represent adduction and abduction moments, respectively

**Figure 4.**
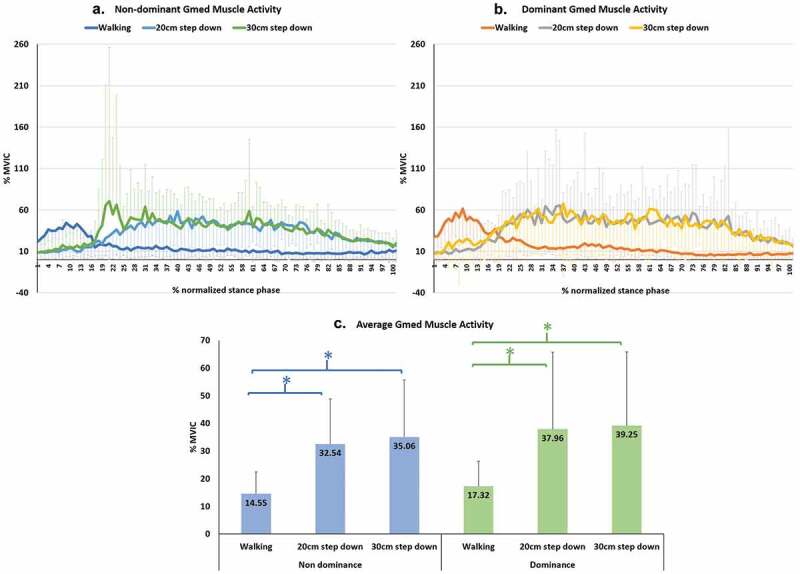
Gluteus medius (GMed) muscle activity during walking and step-down tasks. (a) is the NDLs GMed muscle activity. (b) is the DLs GMed muscle activity. (c) is the multiple comparisons of average GMed muscle

All participants were right leg dominant and age, BMI, and hip strength are reported in Table 1. Pelvic drop to the opposite side and pelvic obliquity excursion were significantly higher in walking than in both step-down tasks (F (1.28, 24.328) = 5.795, p = 0.018, and F (1.058, 20.109) = 24.937, p < 0.001), as was lateral trunk bending excursions (F (1.111, 21.104) = 40.428, p < 0.001). In addition, significantly greater knee adduction moments (F (1.349, 25.635) = 16.671, p < 0.001) were observed. Friedman test showed a significant difference in GMed activity (p < 0.001) with a Chi-square value of 28.526 between functional tasks. Greater average GMed activity (p < 0.05) was seen during both step-down tasks compared to level walking. No significant difference was seen in the clinical hip abductor strength measurements between dominant and non-dominant sides. However, a significant interaction between side and task was seen for hip adduction moment (F (2, 38) = 12.585, p < 0.001), with step-down tasks showing a lower hip adduction moment than walking. All significant main effects are presented in Table 2, and pairwise comparisons between tasks are shown in Table 2 and Figure 4, and the ensemble graphs of kinematics, kinetics, and GMed muscle activity are shown in Figures 2–4.

**Table 1.** The data of participants in the study

N = 20	Mean	SD	95% CI
Male	12		
Female	8		
Age (yr)	30.9	7.0	27.6–34.2
BMI (kg/m^2^)	25.01	3.87	23.23–26.85
NL hip abductor strength (Nm/kg)	1.63	0.29	1.49−1.76
DL hip abductor strength (Nm/kg)	1.61	0.30	1.47–1.75

**Table 2.** Statistical analysis of limb and task effects on biomechanical parameters during the stance of dominant and non-dominant limbs in walking, 20 cm step down, and 30 cm step down

	Task		Pelvic drop to opposite side (degree)	Pelvic obliquity excursion (degree)	Lateral trunk bending excursion (degree)	Peak knee adduction moment (Nm/kg)	Peak hip adduction moment (Nm/kg)
Non-dominant	Walking	Mean	4.9	9.3^*,***^	15.0^*,***^	0.58^*,***^	1.00^****,*,***^
		SD	1.1	3.4	4.4	0.13	0.16
		95% CI	4.3–5.4	7.7–10.9	12.9–17.1	0.52–0.64	0.93–1.07
	20 cm step	Mean	4.0^**^	5.3**	7.1**	0.67**	0.86**
		SD	2.0	1.5	2.5	0.16	0.09
		95% CI	3.0–4.9	4.6–6.0	5.9–8.2	0.60–0.75	0.82–0.90
	30 cm step	Mean	4.8	5.9	8.7	0.70	0.82
		SD	1.9	1.5	2.5	0.19	0.09
		95% CI	3.9–5.7	5.3–6.6	7.5–9.9	0.61–0.79	0.77–0.86
Dominant	Walking	Mean	4.6	9.2^*,***^	14.6^*,***^	0.55^*,***^	1.12^****,*,***^
		SD	1.2	3.4	4.3	0.12	0.16
		95% CI	4.0–5.1	7.7–10.8	12.6–16.6	0.49–0.60	1.04–1.2
	20 cm step	Mean	3.7**	5.0**	6.7**	0.65**	0.87
		SD	1.8	1.6	2.6	0.13	0.10
		95% CI	2.9–4.6	4.3–5.8	5.5–8.0	0.59–0.70	0.83–0.92
	30 cm step	Mean	4.9	6.1	8.6	0.71	0.85
		SD	1.8	1.9	3.1	0.15	0.07
		95% CI	4.1–5.8	5.2–7.0	7.1–10.0	0.64–0.78	0.82–0.89
	Main effect ANOVA p-values	Side	0.696	0.908	0.443	0.478	0.016
		Task	0.018	<0.001	<0.001	<0.001	<0.001
		Interaction	0.403	0.552	0.933	0.486	<0.001

*Significant difference of comparison between walking and 30 cm step tasks (p < 0.05).

**Significant difference of comparison between 20 cm and 30 cm step tasks (p < 0.05).

***Significant difference of comparison between walking and 20 cm step tasks (p < 0.05).

****Significant difference of comparison between non-dominant and dominant (p < 0.05).

## Discussion

In clinical assessment, level walking and step-down tasks are typically used to observe movement symmetry and to determine if any abnormal movements exist. Data from healthy individuals is crucial for understanding the normal movement in order to determine clinically important differences between healthy subjects and people who may be at risk of injury. The purposes of this study were to explore the differences in a clinical assessment of hip strength, GMed muscle activity, coronal plane movements of the trunk and pelvis and moments in the hip and knee joints during walking and step-down tasks, and to determine if any side-to-side differences exist between limbs. All participants in the current study were healthy.

The tasks chosen in this study were common activities of daily living and aimed to give incremental challenges to knee, hip, and trunk control. All parameters were focused on single-limb stance; however, the functional purpose of the single-limb stance during walking and step down is different. During walking the stance limb accepts the body weight from foot contact to push-off phase in order to move the body in a forward direction, whilst during step down the function of the single-limb stance is to control the body descent and allow forward progression without collapsing. Typically, the knee and hip show greater closed-chain ranges of motion during step down than during walking (Andriacchi et al. 1980). Difficulty with walking and stair climbing is typically reported in older adults with knee OA (Guccione et al. 1994; Murray et al. 2013), with the greatest difficulty and knee loading reported during the descending phase of stair climbing (Kaufman et al. 2001).

The pelvic obliquity showed different movements between walking and step-down tests. In the first 50% of stance phase of walking, the pelvis dropped on the opposite side while during step-down tests an ipsilateral pelvic drop was seen (Figure 2). Contralateral pelvic drop occurred within the first 50% of stance phase during walking and the last 50% of stance phase during the step-down tests. This represents differences in the control strategies of pelvic motion in different tasks even though walking and step down are both closed-chain kinetic tasks.

No interaction effects were seen between side and task for trunk, pelvis, and knee movements and knee moments. Task effects significantly influenced pelvic drop to the opposite side, pelvic obliquity excursion, and lateral trunk bending excursion. Approximately 3 to 4 degrees of contralateral pelvic drop were observed during walking and step-down tasks (Table 2). Surprisingly, in pelvic obliquity and lateral trunk bending excursions through stance phase, walking exhibited significantly higher values than the step-down tasks. In the current study, pelvic obliquity excursions were 9.3 degrees during walking, 5.1 degrees during the 20 cm step-down task, and 6.0 degrees during the 30 cm step-down task. Significantly greater amounts of pelvic obliquity excursion were seen during walking than were noted during the 20 cm and 30 cm step-down tasks, 4.2 and 3.3 degrees, respectively. Chockalingam et al. (2012) studied pelvic kinematics in 14 healthy individuals during overground and treadmill walking. Less pelvic obliquity was seen during treadmill walking when compared with overground walking. They suggested that the use of a treadmill could contribute to the change in pelvic range of movement. They reported an average pelvic drop during overground walking of 6.04 degrees and 4.93 degrees for females and males, respectively.

Previous work suggests that lateral trunk bending is related to frontal plane knee moments (Powers 2010). Trunk lateral bending excursions were 14.8 degrees during walking, 6.9 degrees during the 20 cm step-down task, and 8.6 degrees during the 30 cm step-down task. Significantly greater lateral trunk bending excursion was noted during walking when compared to the 20 cm and 30 cm step-down tasks, with a mean difference of 7.9 and 6.2 degrees, respectively. Lateral trunk motion has been associated with knee pain in people with knee OA (van der Esch et al. 2011). However, the relationship between lateral trunk motion and knee pain was weak and was also dependent on age, gender, stiffness, and maximum walking speed. van der Esch et al. reported an average lateral trunk motion of 22.7 degrees. The current study indicates that, in healthy adults, less excursion of pelvic obliquity and lateral trunk bending should be expected during step-down when compared to walking due to the need for greater stability due to the increase in balance challenge.

Knee adduction moment is associated with the loading of the medial compartment of the knee and has been suggested as an important factor in the progression of knee OA (Zhao et al. 2007; Bennell et al. 2011). Knee adduction moment was significantly different between tasks (P < 0.001); however, no side-to-side differences were seen. Peak knee adduction moment occurred within the first 50% of stance phase during walking and occurred within the second 50% of stance phase during the step-down tasks (Figure 3). Few studies have reported the normal range of frontal plane moments during locomotion. Kowalk et al. (1996) studied the frontal plane knee moments during stair climbing (20.3 cm height) in 10 adults (22–40 yrs) and reported that knee abduction moment was 48.25 Nm and 43.81 Nm for the first and second steps, respectively. In this current study we found average peak knee adduction moments of 0.71 Nm/kg, 0.66 Nm/kg, and 0.56 Nm/kg in the 30 cm, 20 cm step-down task and walking, respectively. This indicates that, in healthy adults, the 20 cm and 30 cm step-down tasks produced 15% and 21% greater loading on the medial knee than during walking.

Electromyography is typically used to evaluate the levels of muscle activity which represents the force and work done within the muscle (Richards J. 2008). Figure 4 shows the GMed muscle activity on the NDL and DL normalized to 100% of stance phase. The greatest GMed muscle activity during walking was seen during early stance phase whereas in step down the greatest activity was seen during mid to late stance phase, with greater activity seen in the step-down tasks, with the 30 cm and 20 cm step down on the NDL showing 58.5% and 55.3% greater GMed activity, and the DL showing 55.9% and 54.4% greater activity, respectively, when compared to level walking. This would indicate that, in healthy adults, the controlled movement during step-down tasks requires more GMed activity than during walking, irrespective of the greater hip adduction moment seen during walking.

This study highlights the importance of GMed strength in the control of the hip and pelvis during step-down, which should demonstrate less pelvic excursion than level walking. Moreover, in observational assessments, health professionals should expect to find less excursion of lateral trunk bending in step-down tasks compared to level walking, and represents the typical movement in healthy adults.

A significant interaction effect between side and task was seen for hip adduction moment (p < 0.001) (Table 2), with greater peak hip adduction moments being more apparent in the DL than NDL sides, and walking requiring a higher mechanical demand than both the 20 cm and 30 cm step-down tasks (Table 2). Costigan et al. (2002) found higher peak hip adduction moments during walking (0.95 Nm/kg) when compared to stair ascent (0.8 Nm/kg). In addition, lower knee adduction moments were observed when compared to hip adduction moment for stair ascent (0.42 Nm/kg) and walking (0.49 Nm/kg). Albeit for stair ascent, this supports the findings of this current study which showed that peak hip adduction moments were greater in walking than in step-down tasks and peak hip moments were higher than knee moments in all tasks. It is possible that, in a kinetic chain of normal movements, peak mechanical demand of hip adduction moment would be higher than knee adduction moment. Based on the anatomy of knee and hip joints, there is no muscle around the knee joint which dominantly works to control the frontal plane movement compared to the hip. Therefore, the hip joint has greater involvement with the control in the frontal plane than the knee joint. Previous studies have reported less hip adduction moment in people with moderate to severe knee OA compared to a control group (Astephen et al. 2008). This has led to a proposed hypothesis of decreased hip adduction moment as a result of hip abductor muscle weakness being an important factor in the progression of knee OA (Chang et al. 2005; Mundermann et al. 2005) and has been used as a predictive factor in subgroups in people with patellofemoral pain (Selfe et al. 2016). Small but non-significant differences were seen in hip abductor strength between limbs (1.2%), with an average hip abductor strength of 1.63 Nm/kg for NDL and 1.61 Nm/kg for DL, which represents symmetry in the hip abductor strength between NDL and DL. This study indicates that the clinical strength tests in healthy adults show little difference in hip abductor strength between sides. Previous studies suggest that a 10% and 15%-20% difference in strength between limbs is the cut off to determine asymmetry in athletes and elders, respectively (Augustsson et al. 2004; Perry et al. 2007). However, these have mainly focused on knee strength by using an isokinetic dynamometer. The hip abductor strength test in this study was normalized by thigh length and body weight which allows the strength data to be compared with other studies. Hip abductor strength was measured using a hand-held dynamometer which is relatively inexpensive and can be used in clinical practice. A side-lying position was adopted for the strength test in this study. Widler et al. (2009) reported that side lying is the most appropriate position to record the maximum GMed strength rather than using supine and standing positions, this technique was also used by Selfe et al. (2016) to define clinical subgroups in people with patellofemoral pain. The patterns of lateral trunk bending, pelvic obliquity, knee adduction moment, and GMed muscle activity were all similar between sides; therefore, this study showed no asymmetries in the measurements taken between the NDL and DL. In addition to the use of the 95% CI of hip abductor strength (Table 1), a value of 1.4 Nm/kg may be an appropriate threshold to determine hip abductor weakness when considering rehabilitation in adults. To the authors’ knowledge, there is no evidence of normative data reporting hip abductor strength; therefore, this study provides a useful guide to clinicians to set a minimal goal during rehabilitation in adults with hip abductor weakness. However, a sample size of 20 participants is not sufficient to provide definitive normative data, and further studies are required to confirm these findings and to provide a larger sample size of normative data.

In observational assessments, less excursion of lateral trunk bending in step-down tasks should be expected when compared to level walking. This represents the typical movement in healthy adults. These findings could help clinical professionals to understand the typical movement strategies, which may be useful when comparing individuals with abnormal lower-limb movements. However, it should be noted that the data presented was from a sample of younger adults and clinical professionals should use these values with caution when considering different age groups, although the symmetry of biomechanical parameters may be appropriate in the assessment of older individuals, this requires further work to confirm this assumption.

## Conclusion

The need for greater stability during step down might be the reason why lateral trunk bending and pelvic obliquity excursions exhibited higher values during walking than during the step-down tasks. The controlled descent performed by the stance limb during step-down tasks created a greater knee adduction moment than walking which was also related to step height. Higher peak hip adduction moments were noted in the DL than the NDL sides. However, walking required a greater mechanical demand with greater peak hip adduction moments than the 20 cm and 30 cm step-down tasks, although considerably greater muscle activity was required by GMed to control the hip during the step-down tasks. These findings increase our understanding of the interaction between trunk and pelvic control, and hip and knee joint moments and muscle activity during walking and step-down tasks. This could help clinical professionals to understand the relative demands of the joints and the muscle activity of GMed during these functional tasks. In observational assessments, less excursion of lateral trunk bending should be expected in step-down tasks compared to level walking.

## References

[cit0001] Andriacchi TP, Andersson GB, Fermier RW, Stern D, Galante JO. 1980. A study of lower-limb mechanics during stair-climbing. J Bone Joint Surg Am. 62(5):749–757. 7391098

[cit0002] Arnold CM, Warkentin KD, Chilibeck PD, Magnus CR. 2010. The reliability and validity of handheld dynamometry for the measurement of lower-extremity muscle strength in older adults. J Strength Cond Res. 24(3):815–824. 19661831 10.1519/JSC.0b013e3181aa36b8

[cit0003] Astephen JL, Deluzio KJ, Caldwell GE, Dunbar MJ, Hubley-Kozey CL. 2008. Gait and neuromuscular pattern changes are associated with differences in knee osteoarthritis severity levels. J Biomech. 41(4):868–876. 18078943 10.1016/j.jbiomech.2007.10.016

[cit0004] Augustsson J, Thomee R, Karlsson J. 2004. Ability of a new hop test to determine functional deficits after anterior cruciate ligament reconstruction. Knee Surg Sports Traumatol Arthrosc. 12(5):350–356. 15138668 10.1007/s00167-004-0518-4

[cit0005] Bell AL, Pedersen DR, Brand RA. 1990. A comparison of the accuracy of several hip center location prediction methods. J Biomech. 23:617–621. 2341423 10.1016/0021-9290(90)90054-7

[cit0006] Bennell KL, Bowles KA, Wang Y, Cicuttini F, Davies-Tuck M, Hinman RS. 2011. Higher dynamic medial knee load predicts greater cartilage loss over 12 months in medial knee osteoarthritis. Ann Rheum Dis. 70(10):1770–1774. 21742637 10.1136/ard.2010.147082

[cit0007] Chang A, Hayes K, Dunlop D, Song J, Hurwitz D, Cahue S, Sharma L. 2005. Hip abduction moment and protection against medial tibiofemoral osteoarthritis progression. Arthritis Rheum. 52(11):3515–3519. 16255022 10.1002/art.21406

[cit0008] Chockalingam N, Chatterley F, Healy AC, Greenhalgh A, Branthwaite HR. 2012. Comparison of pelvic complex kinematics during treadmill and overground walking. Arch Phys Med Rehabil. 93(12):2302–2308. 22365476 10.1016/j.apmr.2011.10.022

[cit0009] Costigan PA, Deluzio KJ, Wyss UP. 2002. Knee and hip kinetics during normal stair climbing. Gait Posture. 16(1):31–37. 12127184 10.1016/s0966-6362(01)00201-6

[cit0010] Dierks TA, Manal KT, Hamill J, Davis IS. 2008. Proximal and distal influences on hip and knee kinematics in runners with patellofemoral pain during a prolonged run. J Orthop Sports Phys Ther. 38(8):448–456. 18678957 10.2519/jospt.2008.2490

[cit0011] Distefano LJ, Blackburn JT, Marshall SW, Padua DA. 2009. Gluteal muscle activation during common therapeutic exercises. J Orthop Sports Phys Ther. 39(7):532–540. 19574661 10.2519/jospt.2009.2796

[cit0012] Flack NA, Nicholson HD, Woodley SJ. 2014. The anatomy of the hip abductor muscles. Clin Anat. 27(2):241–253. 23625344 10.1002/ca.22248

[cit0013] Guccione AA, Felson DT, Anderson JJ, Anthony JM, Zhang Y, Wilson PW, Kelly-Hayes M, Wolf PA, Kreger BE, Kannel WB. 1994. The effects of specific medical conditions on the functional limitations of elders in the Framingham study. Am J Public Health. 84(3):351–358. 8129049 10.2105/ajph.84.3.351 PMC1614827

[cit0014] Hewett TE, Torg JS, Boden BP. 2009. Video analysis of trunk and knee motion during non-contact anterior cruciate ligament injury in female athletes: lateral trunk and knee abduction motion are combined components of the injury mechanism. Br J Sports Med. 43(6):417–422. 19372088 10.1136/bjsm.2009.059162 PMC4062295

[cit0015] Kaufman KR, Hughes C, Morrey BF, Morrey M, An KN. 2001. Gait characteristics of patients with knee osteoarthritis. J Biomech. 34(7):907–915. 11410174 10.1016/s0021-9290(01)00036-7

[cit0016] Kim Y, Richards J, Lidtke RH, Trede R. 2018. Characteristics of clinical measurements between biomechanical responders and non-responders to a shoe designed for knee osteoarthritis. Gait Posture. 59:23–27. 28985577 10.1016/j.gaitpost.2017.09.038

[cit0017] Kowalk DL, Duncan JA, Vaughan CL. 1996. Abduction-adduction moments at the knee during stair ascent and descent. J Biomech. 29(3):383–388. 8850644 10.1016/0021-9290(95)00038-0

[cit0018] Mundermann A, Dyrby CO, Andriacchi TP. 2005. Secondary gait changes in patients with medial compartment knee osteoarthritis: increased load at the ankle, knee and hip during walking. Arthritis Rheum. 52(9):2835–2844. 16145666 10.1002/art.21262

[cit0019] Murray CJ, Richards MA, Newton JN, Fenton KA, Anderson HR, Atkinson C, Bennett D, Bernabé E, Blencowe H, Bourne R, et al. 2013. UK health performance: findings of the global burden of disease study 2010. Lancet. 381(9871):997–1020. 23668584 10.1016/S0140-6736(13)60355-4

[cit0020] Perry MC, Carville SF, Smith IC, Rutherford OM, Newham DJ. 2007. Strength, power output and symmetry of leg muscles: effect of age and history of falling. Eur J Appl Physiol. 100(5):553–561. 16847676 10.1007/s00421-006-0247-0

[cit0021] Powers CM. 2003. The influence of altered lower-extremity kinematics on patellofemoral joint dysfunction: a theoretical perspective. J Orthop Sports Phys Ther. 33(11):639–646. 14669959 10.2519/jospt.2003.33.11.639

[cit0022] Powers CM. 2010. The influence of abnormal hip mechanics on knee injury: a biomechanical perspective. J Orthop Sports Phys Ther. 40(2):42–51. 20118526 10.2519/jospt.2010.3337

[cit0023] Richards J. 2008. Biomechanics in clinic and research. London: Churchill Livingstone.

[cit0024] Selfe J, Janssen J, Callaghan M, Witvrouw E, Sutton C, Richards J, Stokes M, Martin D, Dixon J, Hogarth R, et al. 2016. Are there three main subgroups within the patellofemoral pain population? A detailed characterisation study of 127 patients to help develop targeted intervention (TIPPs). Br J Sports Med. 50(14):873–880. 26834185 10.1136/bjsports-2015-094792 PMC4975826

[cit0025] SENIAM. The European recommendations for surface electromyography. [accessed 2017 Jun 1]. http://www.seniam.org.

[cit0026] Tanaka K, Miyashita K, Urabe Y, Ijiri T, Takemoto Y, Ishii Y, Ochi M. 2008. Characteristics of trunk lean motion during walking in patients with symptomatic knee osteoarthritis. Knee. 15(2):134–138. 18255298 10.1016/j.knee.2007.12.009

[cit0027] van der Esch M, Steultjens MP, Harlaar J, van den Noort JC, Knol DL, Dekker J. 2011. Lateral trunk motion and knee pain in osteoarthritis of the knee: a cross-sectional study. BMC Musculoskelet Disord. 12:141. 21714891 10.1186/1471-2474-12-141 PMC3142539

[cit0028] Widler KS, Glatthorn JF, Bizzini M, Impellizzeri FM, Munzinger U, Leunig M, Maffiuletti NA. 2009. Assessment of hip abductor muscle strength. A validity and reliability study. J Bone Joint Surg Am. 91(11):2666–2672. 19884441 10.2106/JBJS.H.01119

[cit0029] Zhang S, Derrick TR, Evans W, Yu YJ. 2008. Shock and impact reduction in moderate and strenuous landing activities. Sports Biomech. 7(2):296–309. 18610780 10.1080/14763140701841936

[cit0030] Zhao D, Banks SA, Mitchell KH, D’Lima DD, Colwell CW Jr, Fregly BJ. 2007. Correlation between the knee adduction torque and medial contact force for a variety of gait patterns. J Orthop Res. 25(6):789–797. 17343285 10.1002/jor.20379

